# Impact of Mediterranean Dietary Intervention on Reactive Oxygen Species Levels and Total Antioxidant Capacity During Pregnancy

**DOI:** 10.3390/cimb47110916

**Published:** 2025-11-04

**Authors:** Eirini Kontopidou, Ioannis Tsamesidis, Areti Kourti, Apostolos Athanasiadis, Aikaterini Itziou

**Affiliations:** 1 Department of Midwifery, School of Health Sciences, University of Western Macedonia, 50200 Ptolemaida, Greece; dmw00009@uowm.gr; 2 Department of Prosthodontics, Faculty of Health Sciences, School of Dentistry, Aristotle University of Thessaloniki, 54124 Thessaloniki, Greece; johntsame@gmail.com; 3 Laboratory of Biochemistry, AHEPA University Hospital, School of Medicine, Aristotle University of Thessaloniki, 54636 Thessaloniki, Greece; aretikourti@auth.gr; 4 Third Department of Obstetrics and Gynaecology, School of Medicine, Faculty of Health Sciences, Aristotle University of Thessaloniki, 54636 Thessaloniki, Greece

**Keywords:** diet, ROS, TAC, oxidative stress, biomarkers, pregnancy, dietary counseling

## Abstract

This study focused on investigating the effects of dietary counseling on antioxidant intakes and how diet can influence reactive oxygen species (ROS) levels and consequently oxidative stress (OS) during pregnancy. At the end of the first trimester of pregnancy (12–13 week of pregnancy), 80 women were randomized into a control group and to an intervention group to receive individual dietary counseling. In the intervention group (n = 60), the dietary counseling about the Mediterranean diet (MD) was carried out during six online meetings every 15 days and focused on antioxidant nutrients and their intake. They were encouraged to follow the antioxidant-rich diet for 12 weeks, including increased consumption of antioxidant-rich foods such as ≥5 servings/day of vegetables, ≥2 servings/day of fruit, ≥8.5 servings/day of wholegrains and 3–4 servings/week of lean meat. In the control group (n = 20) dietary counseling included generic, standard of care guidance about nutrition in pregnancy and was only discussed during a single session. OS biomarkers, particularly (ROS) and Total Antioxidant Capacity (TAC) levels, were analyzed before and after the study duration in women’s serum. The results of the study showed lower ROS and higher TAC levels after intervention compared to the levels before intervention, as well as compared to the levels of the control group. In conclusion, dietary counseling improved the intake of antioxidant nutrients from food during pregnancy, as depicted by oxidative stress biomarkers’ levels, and may influence the observed impacts of these dietary patterns on pregnancy outcomes.

## 1. Introduction

Throughout pregnancy, the body naturally generates reactive oxygen species (ROS) during implantation, proliferation, differentiation and trophoblastic invasion activities, linked to the function of the placenta [1]. Oxidative stress (OS) in pregnancy is mainly caused by the placenta, which is rich in mitochondria [2], and disrupts the mother’s balance from the beginning of pregnancy. As the placenta develops, it transitions from low oxygen levels to high oxygen levels [3]. A significant amount of ROS, such as O_2_^●−^ and NO, are generated due to the high maternal metabolism and activity of mitochondria in the placenta, playing a crucial role in placental blood flow and fetal nutrition [4]. During the first 13 weeks of pregnancy, the placenta has low oxygen levels because it is not yet attached to the mother’s circulation, causing the production of ROS that promote cell growth and the formation of new blood vessels [5], whereas around 12–13 weeks, the placenta is fully developed, resulting in a threefold rise in oxygen levels, causing a rise in ROS levels, predominantly in the syncytiotrophoblast. The placenta also produces nitric oxide locally [6], which, along with other reactive nitrogen species, plays a role in OS when transitional metals are present [7].

Pregnancy is linked to higher vulnerability to OS caused by the body’s inflammatory response [8], a process important for pregnancy, childbirth and the onset of labor [9]. During the third trimester of pregnancy, the systemic inflammatory response activates peripheral white blood cells, resulting in elevated ROS production [10]. Moreover, free radicals are generated when macrophages invade the placenta, producing reactive chlorine species. Myatt and Cui [11] suggest that unbound iron also plays a role in this process [7]. The maternal oxidative balance during pregnancy is affected by various lifestyle factors, including nutritional intake, tobacco use, exercise engagement, environmental contaminant exposure and socioeconomic conditions [12].

OS in pregnancy can affect the mother’s health and hinder the development of the fetus by causing malnutrition and affecting placental blood flow [13], potentially resulting in conditions such as pre-eclampsia and premature labor [14]. In addition, research has also shown that even a regular pregnancy can cause slight OS and result in lipid peroxidation [15]. ROS are responsible for common pregnancy disorders like endothelial cell dysfunction by attacking phospholipids in cell membranes and reacting with fatty acids to produce LPO and damage cells [16]. An overabundance of oxidation can result in oxidative harm at the cellular and biochemical level, affecting various biomolecules like nucleic acids, proteins, lipids and carbohydrates, even in pregnant individuals, although the processes are intricate and necessitate additional study [17].

Human physiology exhibits adaptive redox tolerance mechanisms whereby controlled OS induces the transcriptional activation of supplementary cytoprotective antioxidant pathways. Antioxidant enzymes like catalase (CAT), superoxide dismutase (SOD), glutathione peroxidase, glutathione transferase, glutathione reductase as well as molecules like glutathione collaborate within the cell to convert harmful ROS into harmless substances [18]. However, when OS exceeds physiological defense capacities, it induces macromolecular damage and even cell death.

Maternal nutritional status during gestation significantly influences birth outcomes and long-term offspring’s health [19]. Diets rich in fruits, vegetables, whole grains and unsaturated fats and limited in saturated fats provide bioactive compounds that contribute to antioxidant defenses [19]. Notably, clinical and epidemiological evidence demonstrates that adherence to a Mediterranean diet (MD) correlates with reduced OS and inflammatory biomarkers [20]. Although many dietary interventions have traditionally focused on the effects of one antioxidant nutrient [21], emerging evidence suggests that overall dietary models seem to be more effective in mitigating OS-related complications during the pregnancy period, when pharmacological interventions are often limited. Adherence to healthy dietary patterns like MD during pregnancy is recommended to enhance maternal health and to support proper fetal development by reducing OS levels during the crucial periods of susceptibility. Several micronutrients contribute significantly to antioxidant capacity, like vitamins C and E, selenium, copper and zinc. Selenium is a cofactor of the glutathione peroxidase and thioredoxin reductase, and it is characterized as an antioxidant and anti-inflammatory nutrient. Selenium is essential to producing active thyroid hormones and is essential for normal thyroid function [22]. Longitudinal studies demonstrate a progressive decline in both maternal serum selenium levels and glutathione peroxidase (GPx) activity across gestational trimesters [23]. Clinical evidence indicates that reduced selenium concentrations impair antioxidant defense mechanisms, leading to the compromised protection of biological membranes and DNA during critical phases of embryogenesis [24]. Other studies have shown that low serum selenium concentrations are observed in women with preeclampsia [25]. Although there are few controlled trials on selenium supplementation during pregnancy, some studies have reported that a selenium supplement reduces the incidence of preeclampsia and gestational hypertension [26,27]. Copper also plays an important role in metabolic and antioxidant functions through its involvement in Cu/Zn-SOD activity and facilitates metabolic and antioxidant functions during embryogenesis [28]. During pregnancy, plasma copper concentrations significantly increase, returning to normal nonpregnant values after delivery [29]. A lack of proper nutrition in mothers can lead to immediate effects, such as the early loss of embryos and visible developmental defects, as well as long-term consequences including elevated cardiovascular morbidity and compromised reproductive capacity [30]. Copper, as a redox-active transition metal capable of participating in single-electron transfer reactions, can catalyze the formation of ROS, including harmful hydroxyl radicals. This pro-oxidant activity is thought to contribute to the OS observed in preeclampsia [31]. Zinc is another indispensable trace element regulating DNA synthesis, cellular division and antioxidant protection. All these functions establish zinc’s essential role in successful embryogenesis [32]. During pregnancy, zinc plays critical roles in fetal neurodevelopment and participates in maternal physiological processes during labor [33]. During the third trimester, zinc demand increases to approximately twice that of non-pregnant women [34]. However, nutritional analyses indicate that typical dietary zinc intake among pregnant women meets ≤50% of the recommended daily requirements [35]. This deficiency increases significant risks, as zinc homeostasis disruption is associated with adverse pregnancy outcomes, including prolonged labor, fetal growth restriction (FGR) and fetal death [36]. Clinical studies demonstrate that zinc supplementation reduces the incidence of pregnancy complications, particularly pregnancy-induced hypertension and low birth weight [37]. Notably, fetal zinc concentrations consistently exceed maternal levels, even in preeclamptic pregnancies, suggesting robust fetal zinc regulatory mechanisms independent of maternal status [38].

Vitamins C and E further complement the body’s antioxidant defenses, acting respectively as water- and lipid-phase antioxidants that neutralize ROS and protect cellular membranes from lipid peroxidation [39,40]. However, recent multicenter double blinded randomized trials have demonstrated that combined vitamin C and E supplementation fails to significantly reduce the incidence of preeclampsia or gestational hypertension compared to placebo controls [41]. In addition, dietary phytochemicals such as carotenoids (beta carotene, alpha carotene, lutein, beta kryptoxanthin and lycopene), which are found in many vegetables and fruits with orange and green colors, have many antioxidant and anti-inflammatory effects [42]. Carotenoids play an important role in pregnancy outcomes and have a protective effect against pathologies of pregnancy, which are caused by increased OS [6,43]. A cohort study in Norway found that the concentration of carotenoids in the plasma of pregnant women depends on their intakes of fruit and vegetables [44]. Polyphenols (flavonoids such as quercetin and non-flavonoids such as resveratrol and curcumin) occur naturally in fruits, vegetables, seeds and herbs [45,46]. Polyphenols have antioxidant properties, and they can mitigate the possibility of cancer [47], diabetes [48] and obesity [49]. Polyphenols may influence maternal and fetal outcomes, although their role in pregnancy remains less defined [50].

Given the multifactorial nature of oxidative balance and the limitations of isolated nutrient approaches, evaluating the effects of comprehensive dietary patterns during pregnancy is warranted. Therefore, the present study aimed to evaluate OS biomarkers in pregnant women who followed six online sessions focused on Mediterranean dietary intervention, compared to a control group that received no dietary guidance. The findings could support the development of targeted public health strategies for reducing the risk of adverse pregnancy outcomes.

## 2. Materials and Methods

### 2.1. Research Sample and Research Design

A total of 80 pregnant women were recruited for this study during their first visit to a prenatal testing clinic in Thessaloniki, Greece. The age range of pregnant women was 21–40 years. The participants were at the end of the first trimester of pregnancy (12–13 weeks of pregnancy). They were informed about the purpose of the study and informed consent was obtained from each participant. This study was conducted in strict accordance with the ethical principles outlined in the Declaration of Helsinki (1964) and its subsequent amendments [51]. Ethical approval for this study was granted by the Institutional Research Ethics Committee at the University of Western Macedonia (Protocol Approval Reference: 89/19-07-2022). Exclusion criteria included hypertension, diabetes, placenta previa, genital tract infections and multiple pregnancies, as well as alcohol consumption and cigarette smoking. Women were divided into two groups: 60 participants following Mediterranean dietary intervention (study group) and 20 participants following the standard of care (control group). A block randomization with an intervention: control ratio of 3:1 was followed for the study groups. In the intervention group (n = 60), dietary counseling was carried out during online meetings every 15 days and focused on antioxidant nutrients and their intake. During the online meeting, women in the dietary counseling group received individualized nutritional advice by a registered dietitian based on MD with an emphasis on antioxidants nutrients and food which contained them. The dietary counseling aimed to modify women’s dietary intake and increase the number of antioxidants in women’s diet. They were encouraged to follow the antioxidant-rich diet for 12 weeks, including the increased consumption of antioxidant-rich foods such as ≥5 servings/day of vegetables, ≥2 servings/day of fruit, ≥8.5 servings/day of wholegrains and 3–4 servings/week of lean meat. The consumption of these amounts was voluntary. The vegetables and fruits proposed were polyphenol-rich. Also, the participants were encouraged to follow special recipes enriched with spices which have antioxidant properties, like curcumin and ginger. Beyond the online meetings, communication and advice was offered via email and phone when needed. Participant intake was assessed through a 24 h dietary recall performed during each dietetic session. In the control group (n = 20), dietary counseling included generic, standard-of-care guidance about nutrition in pregnancy and was provided only in a single session. The study was registered to ClinicalTrials.gov (Registration Number NCT07214402) (the Initial Release was on 09/24/2025, and the Last Release was on 10/02/2025).

### 2.2. Blood Collection

Eight-hour fasting blood samples of all women were collected in evacuated containers with EDTA and without anticoagulant from the cubitus vein, at rest. The samples were centrifuged immediately in a dark room at 500× *g* for 15 min at 4 °C. After the serum samples were isolated, they were immediately aliquoted and stored at −80 °C until analyzed after specific pretreatments for each assay.

### 2.3. Reactive Oxygen Species (ROS) Analysis

ROS were detected in serum using the cell-permeable, ROS-sensitive probe 2′,7′-dichlorodihydrofluorescein diacetate (H_2_DCFDA), which emits fluorescence at 520 nm when oxidized (excitation at 480 nm). A 0.5 mM stock solution of H_2_DCFDA in DMSO was used, and the probe was incubated for 30 min with human serum samples as previously described [52]. ROS levels were assessed at each designated time point. Fluorescence measurements were taken using a Tecan fluorometer with black 96-well microplates. ROS concentrations were quantified in millimolar (mm) units by referencing a standard curve generated using hydrogen peroxide (H_2_O_2_) concentrations ranging from 0 to 3 mm.

### 2.4. Total Antioxidant Capacity (TAC) Analysis

Trolox equivalent antioxidant capacity methodology was used to evaluate the serum TAC. Antioxidant activity was assayed spectrophotometrically using a TAC Kit (Total Antioxidant Capacity Colorimetric assay kit, produced by Cayman Chemical Co., Ann Arbor, MI, USA). Antioxidants inhibit the oxidation of 2,2′-azino-bis (3-ethylbenthiazoline)-6-sulphonic acid by the ferryl myoglobin-H_2_O_2_ system. The antioxidant concentration was expressed as millimolar Trolox equivalents (mmol/L).

### 2.5. Statistical Analysis

The data were analyzed using IBM SPSS Statistics 19.0. The normality of distribution of data in all groups of pregnant women was determined by the Kolmogorov–Smirnov normality test. Descriptive statistics, presented as means ± standard deviations, were performed. An independent samples *t*-test was conducted to compare the mean baseline levels of ROS between the two groups prior to the intervention, ensuring initial comparability. Baseline non-parametric levels of TAC were compared between the intervention and control groups using the Mann–Whitney U test. The Wilcoxon signed-rank test was used for both variables to compare the two groups after the intervention. Results were considered significant at *p* < 0.05, with effect sizes (Cohen’s d) reported for clinical relevance.

## 3. Results

### 3.1. Recruitment and Enrollment

**Figure 1:**
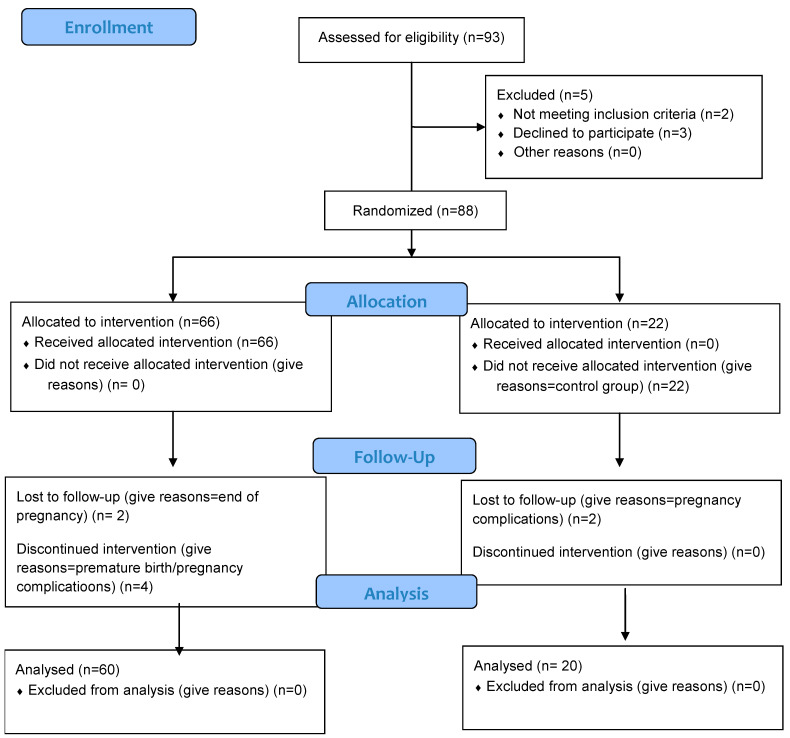
Flow diagram for the study.

A total of 93 pregnant women were enrolled in this study and assessed for eligibility. A total of 5 were excluded, leaving 88 participants eligible for allocation. A total of 6 participants from the intervention group and 2 from the non-intervention group were lost to follow-up, leaving 60 participants from the intervention group and 20 from the non-intervention group for analysis (Figure 1).

### 3.2. Subject Characteristics

The data reveal slight but notable differences between groups. All women were Caucasian and in good health; 45.9% in the intervention group and 31.8% in the control group had a bachelor’s degree. Over half of women (59%) in the intervention group were in its first pregnancy and 55% were in the control group, lived in a city (66.7% vs. 80%) and had a normal body mass index (BMI) (22.79 ± 3.35 vs. 22.36 ± 3.52), as shown in Table 1.

### 3.3. Baseline Comparison of Reactive Oxygen Species (ROS) and Total Antioxidant Capacity (TAC) Analysis

**Figure 2:**
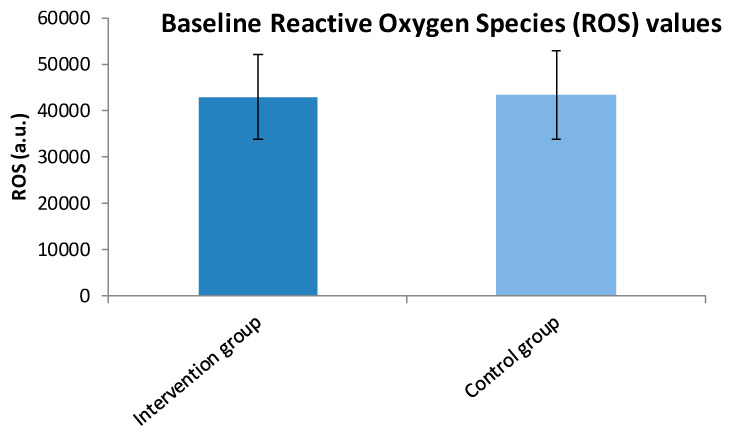
Reactive Oxygen Species (ROS) mean values ± SD in intervention and control groups before intervention.

**Figure 3:**
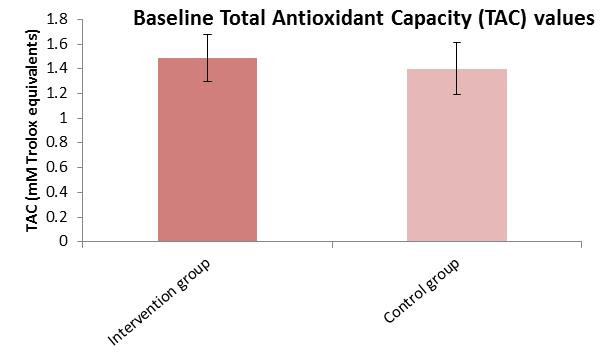
Total Antioxidant Capacity (TAC) mean values ± SD in intervention and control groups before intervention.

An independent samples t-test was conducted to compare the mean ROS values between the intervention and control groups prior to the intervention. There was no significant difference in baseline ROS levels (t (79) = −0.205, *p* = 0.83) between the two groups. The effect size (Cohen’s d = −0.051) indicates a negligible effect, suggesting that both groups were comparable in terms of ROS before the intervention (Table 2) (Figure 2). A Mann–Whitney U test was used to compare TAC scores between the two groups at baseline. Although the intervention group had higher average ranks (43.82), the difference was not statistically significant (*p* = 0.068). The effect size (r = 0.268) suggests a small effect, indicating a slight but non-significant trend toward higher TAC in the intervention group (Table 2) (Figure 3).

### 3.4. Within-Group Analysis of ROS and TAC Variables

**Figure 4:**
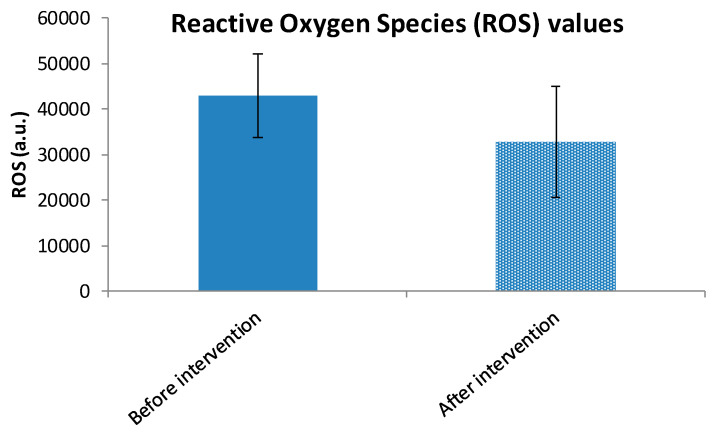
Reactive Oxygen Species (ROS) mean values ± SD before and after intervention in the study group.

**Figure 5:**
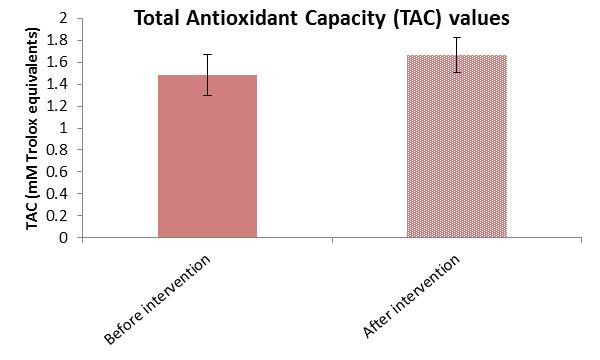
Total Antioxidant Capacity (TAC) mean values ± SD before and after intervention in the study group.

The Wilcoxon Signed-Rank Test was employed to assess differences between paired measurements before and after the intervention, as the data did not meet the assumptions of normality. The analysis revealed a statistically significant decrease in ROS (*p* < 0.001) following the intervention in the study group (before: 42,939.4 ± 9220.8 a.u. vs. after: 32,777.3 ± 12,189.0 a.u.) (Figure 4). The effect size (|r| = 0.723) indicates a large intervention effect, suggesting that the intervention had a strong impact on ROS, supported by the higher mean rank of positive differences (improvement) (30.92) compared to negative differences (28.11). The same statistical test was also used to evaluate changes in TAC before and after the intervention. Despite the negative Z (Z = −4.558) and r values (|r| = 0.675), the results indicate a significant increase in TAC following the intervention (before: 1.48 ± 0.19 mmol/L vs. after: 1.66 ± 0.16 mmol/L) (Figure 5). The intervention had a statistically significant and substantial impact on both variables; it significantly reduced ROS and increased TAC. These findings support the effectiveness of the intervention and highlight the need for further investigation into the mechanisms driving these changes.

**Figure 6:**
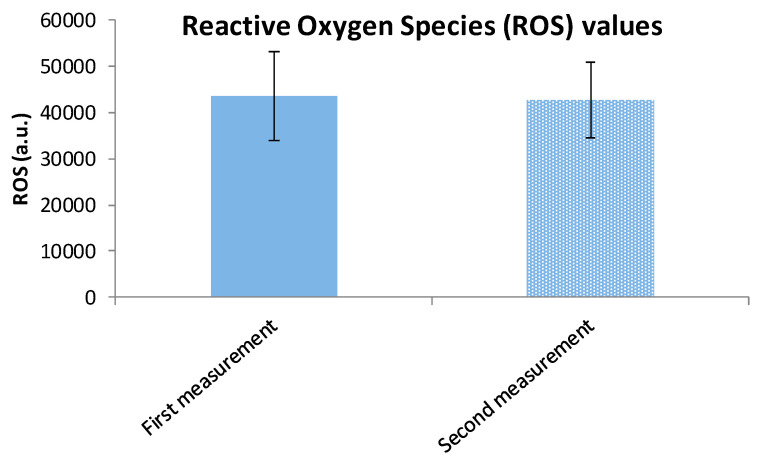
Reactive Oxygen Species (ROS) mean values ± SD in the control group at the first and the second measurement.

**Figure 7:**
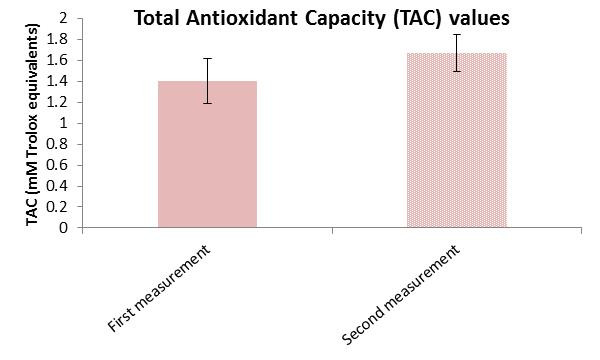
Total Antioxidant Capacity (TAC) mean values ± SD in the control group at the first and the second measurement.

Moreover, in the control group, there was no significant change in ROS levels, which averaged 42,771.5 ± 8175.5 a.u. vs. 43,422.6 ± 9589.1 a.u. before intervention (Figure 6). The similar mean ranks for positive (9.67) and negative (12.78) differences further confirm the absence of meaningful variation. The effect size was essentially zero (|r| = 0.004), indicating that the control condition had no measurable impact on OS. Despite the absence of an active intervention, the control group showed a significant (*p* < 0.001) increase in TAC levels averaged 1.672 ± 0.175 mmol/L vs. 1.401 ± 0.212 mmol/L in the baseline (Figure 7). The effect size (Cohen’s d = −1.10) is considered large, suggesting a substantial change. This result may warrant further investigation into potential confounding factors or natural variability.

ROS levels decreased significantly in the intervention group while remaining nearly unchanged in the control group. TAC levels increased in both groups, but the increase was more pronounced in the intervention group (Table 3).

### 3.5. Endpoint Comparison of Reactive Oxygen Species (ROS) and Total Antioxidant Capacity (TAC) Analysis

The intervention group displayed clinically and statistically significant improvements in both ROS reduction and TAC elevation, with large effect sizes underscoring the intervention’s efficacy (Table 4). The Wilcoxon Signed-Rank Test revealed a statistically significant reduction in ROS levels following the intervention (*Z* = −4.873, *p* < 0.001). The median ROS value decreased from 42,461 (pre-intervention) to 30,538 (post-intervention), indicating a substantial improvement. Similarly, TAC levels showed a statistically significant increase post-intervention (*Z* = −4.558, *p* < 0.001). The median TAC rose from 1.47 (pre-intervention) to 1.71 (post-intervention). The negative r value (−0.675) reflects the inverse relationship between ROS and TAC, with the effect size again indicating a large magnitude of change.

In contrast, the control group’s TAC increase, despite its statistical significance, lacked association with ROS modulation, raising questions about its biological relevance. The control group exhibited no statistically significant change in ROS levels (*Z* = −0.017, *p* = 0.986). Median ROS values remained stable (pre: 44,125; post: 42,857), with a negligible effect size (r = 0.004). A paired samples t-test demonstrated a significant increase in TAC (t = −5.046, *p* < 0.001), with a mean value of 1.67 a.u. (*SD* = 0.17). The large Cohen’s d (−1.10) suggests a strong effect, though this change occurred without a corresponding ROS reduction, potentially indicating external factors influencing TAC independently of the intervention.

## 4. Discussion

A proper diet during pregnancy is crucial for both the mother and the fetus, as it supports maternal health throughout gestation, childbirth and breastfeeding [53]. This study aimed to investigate the effect of the consumption of antioxidant foods like vegetables, fruits, seeds and vegetables oils through dietary counseling in pregnant women on OS biomarkers. These diet changes result in health benefits for the outcome of pregnancy and for the fetus or infants [54]. The results of the present study showed that pregnant women who followed a Mediterranean dietary intervention for 12 weeks exhibited a statistically significant reduction in ROS levels and a significant increase in TAC levels by the end of the intervention compared to baseline. Furthermore, ROS levels were significantly lower and TAC levels were significantly higher in the intervention group compared to the control group.

A nutritionally balanced diet is a primary source of nonenzymatic antioxidants. Diets rich in fruits, vegetables, whole grains and healthy fats—particularly monounsaturated and polyunsaturated fats—and low in saturated fats provide essential bioactive compounds that support the body’s antioxidant defense mechanisms. Numerous observational and clinical studies have demonstrated that adherence to the MD is associated with significant reductions in established biomarkers of OS [20]. Specifically, adherence to the MD has been linked to decreased levels of F2-isoprostanes and enhanced TAC in adults. It has also been associated with reductions in lipid peroxidation and oxidative DNA damage [55]. These beneficial effects are likely due to the MD’s abundance of bioactive nutrients, which strengthen the body’s natural antioxidant systems and also help neutralize free radicals directly [56]. Furthermore, the MD’s positive influence on vascular function and its anti-inflammatory properties may contribute to its ability to lower OS [57]. Several studies have found that a diet rich in antioxidants is associated with the improvement in sperm’s motility in infertile men and can lead to positive pregnancy outcomes [58]. MD supports proper ovulation in women [59] and increases the percentage of success of the pregnancy after undergoing assisted reproduction methods [60]. Specifically, the intake of dietary carotenoids may benefit women with genetic predispositions to spontaneous abortion [61]. While earlier research suggested that adherence to the MD does not significantly reduce the risk of miscarriage [62], more recent evidence indicates that an antioxidant-rich diet may lower miscarriage rates in women with recurrent pregnancy loss [63]. Furthermore, a recent review concluded that a balanced diet incorporating antioxidant-rich foods can contribute to miscarriage prevention [64].

During pregnancy, the levels of antioxidants in a woman’s body increase to balance oxidation [65], and several protective mechanisms arise to counteract the formation and harmful effects of free radicals. However, OS levels depend on the balance between antioxidant defenses and free radical production [18]. Differences in TAC values were depicted between the first and second measurements in the control group of the present study. This indicates lower antioxidant levels in the early pregnancy, while as pregnancy progresses into the second and third trimesters, a woman’s antioxidant capacity increases [8]. According to Hussain et al. [8], TAC activity begins to rise after the eighth week of pregnancy, which may be associated with changes in plasma uric acid levels. Furthermore, dietary patterns may play an important role in oxidation balance. It is possible that the increase in TAC levels in the control group can be additionally due to the minimal advice given, which may have prompted some dietary improvement. Rodríguez-Cano et al. [66] found that consuming ultra-processed foods (UPFs) during pregnancy may raise OS levels and potentially impact pregnancy outcomes. Therefore, nutrition guidance that emphasizes reducing or avoiding UPFs and adopting an MD could help enhance the body’s antioxidant defenses. MD with an emphasis on olive oil and nuts is particularly noteworthy, as these foods contain high levels of monounsaturated fats and antioxidants such as vitamin E and polyphenols, which scavenge free radicals and protect cellular structures [67]. Also, MD is characterized by a daily intake of fruits and vegetables, regular legume and nuts intake and a high intake of virgin olive oil, providing a robust supply of natural antioxidants, including vitamins C and E, polyphenols and carotenoids [68]. Another compound of citrus fruits, grapes, olive oil, onions and wine is flavonoid taxifolin (dihydroquercetin), which has shown an antioxidant effect due to its property of inhibition of oxidative enzymes responsible for ROS overproduction [69]. The most recent experimental study suggests that taxifolin significantly attenuates intracellular ROS generation upon subsequent hydrogen peroxide (H_2_O_2_) challenge [70]. In addition to MD, many antioxidant bioactive compounds are found in edible flowers or medicinal plants [71]. Zingiber officinale is very often used in nutraceuticals for therapeutic use and as a spice for flavoring foods and drinks [72]. Gingerols and shogaols have antioxidant, anti-inflammatory and antiallergic activities [73]. Particularly, the scientific literature has reported bioactive compounds contained in ginger with antioxidant activities in human stem cells [74] and the uterus of rats [75]. Dried ginger has been found to have better results for antioxidant activity compared with fresh ginger [76]. On top of that, antioxidant therapy from dietary sources can complement endogenous antioxidants in restoring redox homeostasis [77]. Some studies highlight that the supplementation of curcumin has beneficial effects on placental oxidative damage in mouse and rat [78]. Moreover, the dietary polyphenol supplementation of pregnant rats with resveratrol and curcumin reduces the oxidative damage in the liver of the offspring [79]. These compounds play a crucial role in counteracting OS, which is elevated during pregnancy due to increased metabolic activity and mitochondrial function.

Moreover, the MD’s anti-inflammatory properties may further contribute to the reduction in OS by downregulating the production of pro-inflammatory cytokines that promote ROS generation. The benefits of MD on OS are not solely limited to maternal health but also extend to fetal development. Higher serum TAC levels in pregnant women following the MD may reflect improved systemic antioxidant defenses. This enhancement can help mitigate oxidative damage to maternal tissues and the developing fetus and plays a pathogenic role in several major pregnancy complications, including preeclampsia, intrauterine growth restriction and preterm birth [80]. According to recent literature data, a pro-inflammatory diet with reduced consumption of antioxidants nutrients may lead to fetal growth restriction [81]. Elevated MD adherence in pregnancy may positively influence intrauterine growth and specific acute and chronic issues related to prematurity, along with maternal hypertension/preeclampsia [82]. Pregnant women with diabetes [GDM] produce excess free radicals and their scavenging mechanisms are deteriorated [83]. When blood glucose is high, the levels of antioxidants like vitamins C and E decrease and the metabolites of oxidases and peroxides increase [84]. OS disrupts glucose regulation using different signal transduction, which leads to the exacerbation of GDM [85]. A study by Assaf-Balut et al. [86] found that pregnant women following a Mediterranean-style diet containing extra virgin olive oil and pistachios had lower rates of gestational diabetes and OS markers, suggesting an improved metabolic and antioxidant status that may influence fetal outcomes. The daily consumption of extra virgin olive oil has been found to reduce the levels of triglycerides in GDM pregnant women and have an anti-inflammatory effect on the placenta [87]. Similarly, a systematic review of intervention studies established that antioxidant supplementation has a possible effect for fasting insulin for women with gestational diabetes [88]. On the other hand, findings from Bernardo et al. [89] suggest that a low-protein diet during pregnancy in rats impairs the antioxidant defense system and may lead to mitochondrial dysfunction. In the same trend, research data support that climate change affects agricultural ecosystems with alterations in the antioxidant capacity of certain crops, like grains and legumes, and reduces the intake of antioxidant nutrients through diet [90].

Vitamins E and C are the most well-known antioxidants nutrients with a prophylactic role of developing preeclampsia during pregnancy. Some researchers found a significant reduction in the risk of developing preeclampsia when there was supplementation with these vitamins compared with the control group [91]. A study involving dietary counseling found that vitamin E intake was below recommended levels. However, the intervention group showed higher intakes, suggesting that dietary counseling can effectively increase antioxidant nutrient intake during pregnancy [92]. Moreover, in the study which assessed the impact of individual dietary counseling in allergic pregnant women, there was a higher intake of vitamin E compared to controls [93]. The same outcome was found in the study among Finnish pregnant women, who increased their vitamin E intake through diet counseling, which had beneficial effects on the total intake of vegetable, fruit berries and cereal products. However, nutritional counseling interventions showed no significant effect on vitamin C intake levels despite targeting the increased consumption of fruits and vegetables, which constituted the major dietary sources of ascorbic acid in this population [94]. Another study using a food-based approach to provide 15 mg of vitamin E through dietary counseling found no significant differences between the control and intervention groups [95]. Mean serum a-tocopherol increased in both groups, as a physiological increase happens from 12 weeks of pregnancy onward [96]. A systematic review and metanalysis found that there was no significant difference in the risk of preeclampsia between pregnant women receiving supplementation with vitamins C and E compared to those to placebo group [97]. The findings from research on pregnancy and congenital heart defects (CHD) showed that there are some indices during pregnancy, such maternal diet, which can affect the prediction of heart diseases [98]. Dietary habits of mothers can influence fetal cardiovascular development, either with antioxidant or antioxidant effects and the increase in ROS [99]. Yang et al. [100], using oxidative balance score (OBS), support that higher OBS during pregnancy can result in lower OS and prevent the incidence of CHD.

Additionally, although existing data are limited and sometimes controversial, research suggests that maternal antioxidant supplementation may help prevent chronic diseases. Recent scientific literature [101] indicates that such supplementation can support overall offspring health. However, nutritional education should be introduced in all pregnancies before either antioxidant supplementation or pharmaceutical intervention is suggested.

The present study provides valuable evidence on the impact of the MD on OS and TAC in pregnancy. However, some limitations should be acknowledged. First, both cases and controls were recruited from only one prenatal testing clinic, which may bring selection bias. Second, pregnant women may not adhere to a typical MD diet or have seasonal variability regarding the intake of fruits and vegetables, although we conducted online meetings every fifteen days and provided constant support. Moreover, underreporting, which is common in pregnant women due to nausea or selective memory, may have occurred [102]. Third, the cohort of the study could be larger and from various cities across Greece to enhance generalizability. Finally, residual confounders from unknown factors may exist.

## 5. Conclusions

While some variability exists in study outcomes due to differences in dietary assessment tools, participant adherence and baseline nutritional status and randomized controlled trials remain limited, observational data consistently point toward a favorable effect of the MD on OS modulation in pregnant populations. In conclusion, our study supports the role of an MD-based intervention in reducing OS and enhancing TAC during pregnancy. This dietary pattern, rich in antioxidants and anti-inflammatory compounds, offers a non-pharmacological strategy for supporting maternal and fetal health. Further longitudinal and interventional studies are needed to confirm causality and explore long-term outcomes. In contrast, women in the control groups who did not follow any structured dietary plan, often consumed diets lower in antioxidant-rich foods, resulting in comparatively lower TAC levels. This disparity supports the hypothesis that targeted dietary strategies can positively influence oxidative balance during pregnancy.

In summary, the present evidence strongly supports that pregnant woman adhering to an antioxidant-rich MD exhibit significantly higher serum TAC levels, highlighting the importance of nutritional guidance during gestation. Our findings suggest that following healthy dietary patterns, such as the antioxidant-rich MD, during pregnancy may support maternal well-being and promote healthy fetal development by lowering OS during this sensitive stage.

## Figures and Tables

**Table 1:** Baseline characteristics of pregnant women by group allocation.

Variable	Intervention Group (n = 60)	Control Group (n = 20)
Age (year)	41 ± 4.01	34.2 ± 4.68
Height (cm)	165.57 ± 5.76	165.7 ± 5.00
Weight before pregnancy (kg)	62.37 ± 8.75	61.50 ± 10.34
Weight at first trimester (kg)	64.50 ± 8.31	66.72 ± 12.00
BMI * (kg/m^2^) before pregnancy	22.79 ± 3.35	22.36 ± 3.52
BMI (kg/m^2^) at first trimester	23.58 ± 3.26	24.27 ± 4.06
Maternal education		
High school [n (%)]	6 (10)	1 (5)
IVT * [n (%)]	5 (8.3)	6 (30)
Bachelor’s degree [n (%)]	28 (46.7)	7 (35)
Master’s degree [n (%)]	17 (28.3)	6 (30)
Ph.D. [n (%)]	4 (6.7)	0 (0)
Urban residence [n (%)]	40 (66.7)	16 (80)
Rural residence [n (%)]	20 (33.3)	4 (20)
Gravidity		
First [n (%)]	35 (58.3)	11 (55)
Second [n (%)]	20 (33.3)	6 (30)
Third [n (%)]	4 (6.7)	2 (10)
Fourth [n (%)]	1 (1.7)	1 (5)
Maternal occupation		
Private sector [n (%)]	25 (41.7)	7 (35)
Public sector [n (%)]	8 (13.3)	3 (15)
Freelancer [n (%)]	18 (30)	7 (35)
Household [n (%)]	4 (6.7)	2 (10)
Unemployed [n (%)]	5 (8.3)	1 (5)

* Values are means ± SDs; BMI: Body Mass Index; IVT = Institutes of Vocational Training.

**Table 2:** Comparative Overview of Statistical Tests for baseline ROS and TAC.

Variable	Mean Rank/Mean	Sum of Ranks/SD *	Test Statistics	*p*-Value
ROS * Intervention Group	42,939.40 a.u.	9220.86 (SD)	t = −0.205	0.838
ROS Control Group	43,422.62 a.u.	9589.19 (SD)	-	-
TAC * Intervention Group	43.82	2629	Z = −1.823	0.068
TAC Control Group	32.95	692	-	-

* ROS: Reactive Oxygen Species; TAC: Total Antioxidant Capacity; SD: Standard Deviation; a.u.: arbitrary unit.

**Table 3:** Within-Group Comparisons for ROS and TAC Variables.

Variable	Direction	N	Mean Rank/Mean	Sum of Ranks/SD *	Test Statistics	*p*-Value
ROS * Intervention Group	Negative Ranks	51	30.92	1577.00	Z = −4.873	<0.001
	Positive Ranks	9	28.11	253.00		
TAC * Intervention Group	Negative Ranks	13	22.77	296.00	Z = -4.558	<0.001
	Positive Ranks	47	32.64	1534.00		
ROS Control Group	Negative Ranks	11	9.67	116.00	Z = −0.017	0.986
	Positive Ranks	9	12.78	115.00		
TAC Control Group	Mean Difference	20	Pre: 1.401	0.212 (SD)	t = −5.046	<0.001
			Post: 1.672	0.175 (SD)		

* ROS: Reactive Oxygen Species, TAC: Total Antioxidant Capacity, SD: Standard Deviation.

**Table 4:** Comparative Overview of Statistical Tests for endpoint ROS and TAC.

Variable	Median/Mean	Test Statistics	*p*-Value
ROS * Intervention Group	30,538	Z = −4.873	<0.001
ROS Control Group	42,857	Z = −0.017	0.986
TAC * Intervention Group	1.71	Z = −4.558	<0.001
TAC Control Group	1.67 mmol/L	t = −5.046	<0.001

* ROS: Reactive Oxygen Species; TAC: Total Antioxidant Capacity.

## Data Availability

The original contributions presented in this study are included in the article. Further inquiries can be directed to the corresponding author.
